# Effectiveness and safety of weekly paclitaxel and cetuximab as a salvage chemotherapy following immune checkpoint inhibitors for recurrent or metastatic head and neck squamous cell carcinoma: A multicenter clinical study

**DOI:** 10.1371/journal.pone.0271907

**Published:** 2022-07-28

**Authors:** Takahiro Wakasaki, Tomomi Manako, Ryuji Yasumatsu, Hirotaka Hara, Satoshi Toh, Muneyuki Masuda, Moriyasu Yamauchi, Yuichiro Kuratomi, Emi Nishimura, Toranoshin Takeuchi, Mioko Matsuo, Rina Jiromaru, Kazuki Hashimoto, Noritaka Komune, Takashi Nakagawa

**Affiliations:** 1 Department of Otorhinolaryngology, Graduate School of Medical Sciences, Kyushu University, Fukuoka, Japan; 2 Department of Head and Neck Surgery, National Hospital Organization, Kyushu Cancer Center, Fukuoka, Japan; 3 Department of Otorhinolaryngology-Head and Neck Surgery, Faculty of Medicine, Kindai University, Osaka, Japan; 4 Department of Otolaryngology, Head and Neck Surgery, Saga University Hospital, Saga, Japan; 5 Department of Otorhinolaryngology, Kitakyushu Municipal Medical Center, Kitakyushu, Japan; University of Nebraska Medical Center, UNITED STATES

## Abstract

**Objectives:**

The benefit of sequential therapy after immune checkpoint inhibitor (ICI) treatment for recurrent or metastatic head and neck squamous cell carcinoma (R/M HNSCC) has been recently reported. Furthermore, there is a growing interest in the impact of cetuximab (Cmab)-containing salvage chemotherapy (SCT) and the therapeutic efficacy and adverse events (AEs) of Cmab administration prior to ICI administration.

**Materials and methods:**

We retrospectively reviewed the medical records of 52 patients with R/M HNSCC treated with SCT (weekly paclitaxel [PTX], n = 7, or weekly PTX and Cmab [PC], n = 45).

**Results:**

The objective response rate (ORR) and a disease control rate (DCR) was 53.3% and 91.1% in the PC group and 42.9% and 57.1% in the PTX group, respectively. There was a significant difference in the DCR between the PC and PTX groups (p = 0.0143). The overall survival (OS) and progression-free survival were significantly better in the PC group than in the PTX group. On the other hand, the incidence of drug-induced interstitial pneumonia (DI-IP) in R/M HNSCC patients who received SCT was 21.2%. Patients in the PC group were divided according to whether they received Cmab (Group A) or did not receive Cmab (Group B) as palliative therapy prior to ICIs. Group B had a significantly better OS than Group A. Furthermore, our findings suggest that the incidence rate of DI-IP during SCT might be higher in Group B.

**Conclusion:**

Although PC following ICIs shows dramatic efficacy, careful monitoring of AEs, including DI-IP, is recommended.

## Introduction

In recent years, immune checkpoint inhibitors (ICIs) have markedly improved treatment outcomes for patients with recurrent or metastatic head and neck squamous cell carcinoma (R/M HNSCC), leading to a paradigm shift in palliative chemotherapy for these patients [1–3]. In particular, long-term survivors have emerged whose performance status (PS) has been maintained [4–6]. Because the number of patients who respond to the immune checkpoint inhibitor nivolumab alone is limited, the importance of sequential chemotherapy strategies is increasingly becoming recognized [7]. We previously reported excellent effectiveness and adverse effects of salvage chemotherapies (SCTs) following ICIs, and combined chemotherapy of weekly paclitaxel and cetuximab (PC) as SCT presented a good response (objective response rate [ORR] 60%, disease control rate [DCR] 96%, n = 25) [8].

On the other hand, DI-IP is one of the most serious adverse events associated with the use of anticancer drugs, and the onset of DI-IP differs between races. The IP prevalence among Japanese populations is has been reported as particularly high compared with the populations of Western countries [9, 10]. Despite the excellent therapeutic effect, we also reported drug-induced interstitial pneumonia (DI-IP) during SCT in 138 patients with R/M HNSCC (incidence rate, 7.2%) [8, 11, 12]. DI-IP was also reported to occur more frequently in patients treated with CP following nivolumab monotherapy (5/20, 25%) than in patients treated with other regimens [11]. In contrast, Kurosaki et al. reported that there were no associations between the incidence rate of DI-IP and the treatment regimens [13]. However, they also mentioned the possibility of selection bias could not be excluded in their study because approximately half of the subjects received prior cetuximab before immunotherapy, which meant that patients who were tolerable to anti-EGFR antibodies in earlier lines of therapy were included. It is still controversial for the occurrence rate of DI-IP and the therapeutic effect in patients with R/M HNSCC who underwent CP following ICI treatment. Therefore, we increased the number of patients from our previous report, and the present study aimed to evaluate the effectiveness and safety of weekly paclitaxel (PTX) with or without Cmab after exposure to ICIs affected by prior cetuximab use.

## Materials and methods

### Patients

**Fig 1:**
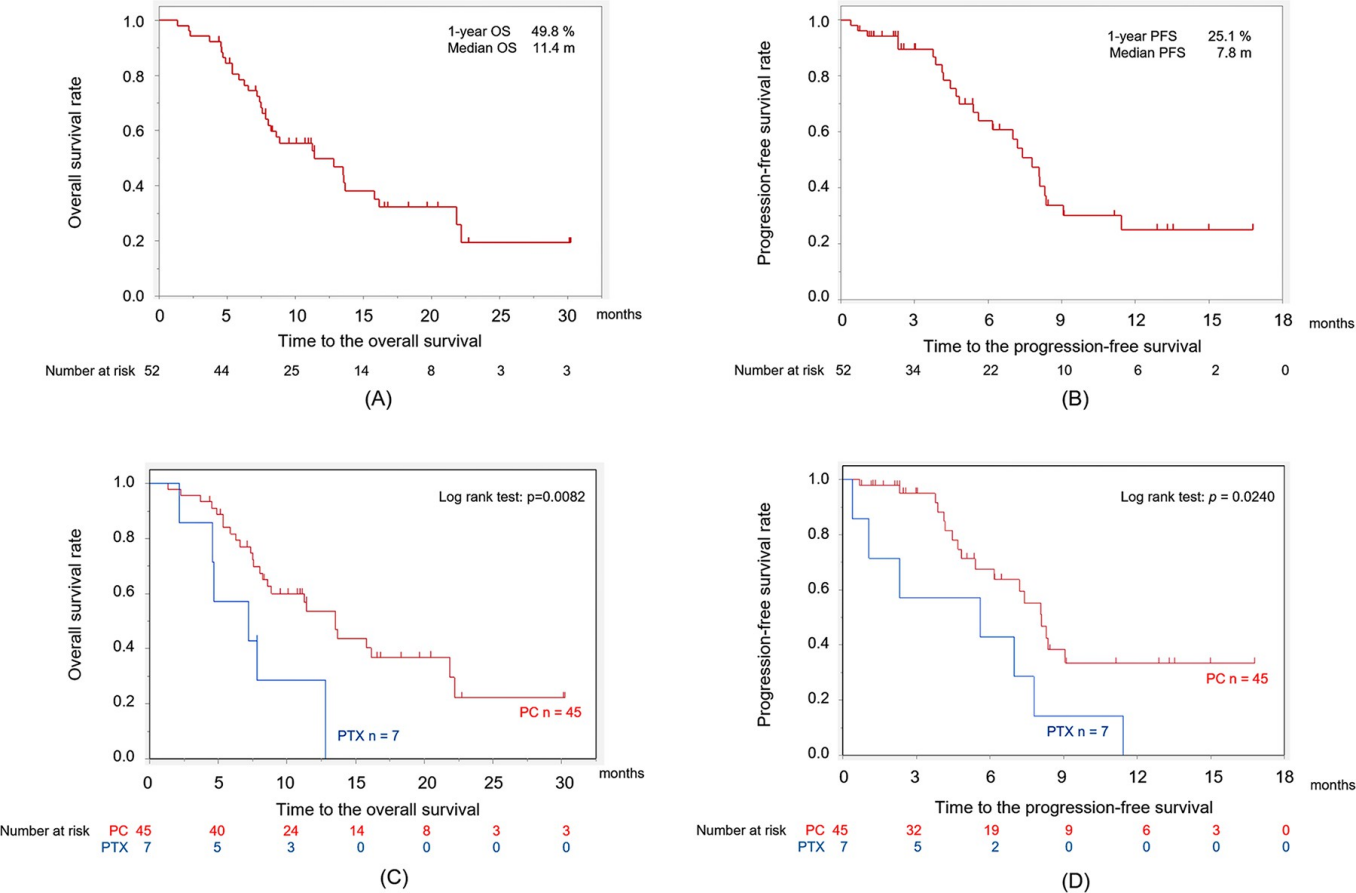
Kaplan–Meier curves for (A) overall survival and (B) progression-free survival in all R/M HNSCC patients who received SCT following nivolumab treatment. There were significant differences in overall survival (C) and progression-free survival (D) between the patients administered PC and those administered PTX (p = 0.0082 and p = 0.0240, respectively). R/M HNSCC, recurrent or metastatic head and neck squamous cell carcinoma; PC, combined chemotherapy of weekly paclitaxel and cetuximab; PTX, paclitaxel; SCT, salvage chemotherapy.

From April 1, 2017, to July 31, 2020, 52 patients with R/M HNSCC were treated with PC or PTX as SCT following nivolumab administration at Kyushu University Hospital, National Kyushu Cancer Center, Saga University Hospital, and Kitakyushu Municipal Medical Center, Japan. All tumors were histologically confirmed as squamous cell carcinoma. When nivolumab administration (3 mg/kg or 240 mg/body every 2 weeks) was terminated because of progressive disease (PD), SCT was subsequently administered as PC (weekly PTX, 80 mg/m^2^, and weekly Cmab, 400 mg/m^2^ for the first week and 250 mg/m^2^ for the subsequent weeks) or PTX (80 mg/m^2^) to 45 and 7 patients, respectively (Fig 1). Chemotherapy regimens were selected according to the status of each patient, which was determined by the Head and Neck Cancer Boards of the respective institutions. The patients received treatment until progression or the development of unacceptable toxicity.

The study protocol was approved by the Institutional Review Board of Kyushu University (reference number: 2021–138) and was conducted in accordance with the principles of the Declaration of Helsinki. All patients gave their written informed consent for study participation. We have opted out of the research content on our institution’s website.

### Evaluation of the response and adverse events

We evaluated the tumor response using the Response Evaluation Criteria in Solid Tumors (version 1.1) based on the findings of computed tomography (CT) imaging, which was performed every 8–12 weeks. PD was defined as a ≥20% increase in the sum of the diameters of the target lesions or the appearance of new metastatic lesions. Stable disease (SD) was defined as ranging from a decrease of <30% to an increase of <20% in tumor size on imaging. A partial response (PR) was defined as a decrease of ≥30% in the sum of the diameters of the target lesions. We evaluated the best overall response of all patients as a complete response (CR), PR, SD, or PD. The ORR corresponded to a CR or PR, and the DCR corresponded to a CR, PR, or SD. OS was defined as the time from the first day of SCT treatment until death, and PFS was assessed to the day of disease progression or death. Toxicity was assessed according to the Common Terminology Criteria for Adverse Events (version 4.0).

### Statistical analyses

All statistical analyses were performed using JMP 16 software (SAS Institute, Cary, NC, USA). Categorical variables were analyzed using the Fisher exact test. Differences with a p-value < 0.05 were considered statistically significant. OS and PFS were calculated using the Kaplan–Meier method and were evaluated with the log-rank test.

## Results

### Patient characteristics

The clinical characteristics of the 52 patients (45 men and seven women; median age, 65 years; range, 33–79 years) are summarized in Table 1. The primary site of the tumor varied and was located in the oral region, oropharynx, hypopharynx, or larynx in 38 patients (73.1%). All tumors were histologically confirmed as squamous cell carcinoma. Distant metastasis with or without locoregional recurrence occurred in 32 patients (61.5%), and 20 (38.5%) of 52 had locoregional recurrence. In these 52 patients, the ORR and DCR of nivolumab were 17.3% and 53.8%, respectively (S1 Table). The Eastern Cooperative Oncology Group PS at the first administration of SCT was 0–1 in 46 patients, 2 in five patients, and 3 in one patient. The patients were treated with PC (45/52, 86.5%) or PTX (7/52, 13.5%) as SCT. The rates of second-, third-, fourth-, and fifth-line treatments were 57.7% (30/52), 34.6% (18/52), 5.8% (3/52), and 1.9% (1/52), respectively. Patient follow-up lasted until death or until the cut-off date (January 31, 2021). The median overall follow-up interval was 9.2 months (range, 1.3–30.2 months).

**Table 1:** Characteristics of the patients included in this study.

	All (n = 52)		PTX (n = 7)		PTX + Cmab (n = 45)	
	n	%	n	%	n	%
**Median Age**		65 (33–79)		65 (33–79)		67 (45–79)
≥65 years old	29	55.8	4	57.1	25	55.6
<65 years old	23	44.2	3	42.9	20	44.4
**Gender**						
Male	45	86.5	7	100.0	38	84.4
Female	7	13.5	0	0.0	7	15.6
**Smoking status**						
Smoker	43	81.1	6	85.7	37	82.2
Never	9	17.0	1	14.3	8	17.8
**ECOG PS at the first administration of SCT**						
PS 0–1	46	88.5	7	100.0	39	86.7
PS 2–3	6	11.5	0	0.0	6	13.3
**Primary site**						
Oral	14	26.9	2	28.6	12	26.7
Nasopharynx	2	3.8	1	14.3	1	2.2
Oropharynx	7	13.5	0	0.0	7	15.6
Hypopharynx	12	23.1	2	28.6	10	22.2
Larynx	5	9.6	1	14.3	4	8.9
Sinonasal tract	9	17.3	1	14.3	8	17.8
Others	3	5.8	0	0.0	3	6.7
**Disease State**						
LA	20	38.5	3	42.9	17	37.8
DM	17	32.7	2	28.6	15	33.3
LA + DM	15	28.8	2	28.6	13	28.9
**Administration line of SCT in palliative therapy**						
2nd	30	57.7	4	57.1	26	57.8
3rd	18	34.6	3	42.9	15	33.3
4th	3	5.8	0	0.0	3	6.7
5th	1	1.9	0	0.0	1	2.2
**Cause of cessation of Nivolumab**						
PD	48	92.3	42	93.3	6.0	85.7
AE	1	1.9	1	2.2	0.0	0.0
PD+AE	1	1.9	0	0.0	1.0	14.3
Other reason	2	3.8	2	4.4	0.0	0.0
Ongoing	0	0.0	0	0.0	0.0	0.0
**Cause of cessation of SCT**						
PD	20	38.5	16	35.6	4.0	57.1
AE	25	48.1	22	48.9	3.0	42.9
PD+AE	1	1.9	1	2.2	0.0	0.0
Other reason	3	5.8	3	6.7	0.0	0.0
Ongoing	3	5.8	3	6.7	0.0	0.0

AE, adverse event; Cmab, cetuximab; DM, distant metastasis; PS, performance status; ECOG, Eastern Cooperative Oncology Group; LA, locally advanced disease; PD, progressive disease; PS, performance status; PTX, paclitaxel; SCT, salvage chemotherapy

### Overall treatment efficacy of salvage chemotherapy

The ORR was 51.9% (27/52) and DCR was 86.5% (45/52) in patients treated with chemotherapy after nivolumab treatment (Table 2). Furthermore, the ORR and DCR in the patients treated with PC were 53.3% and 91.1%, respectively, and 42.9% and 57.1% in the patients treated with PTX, respectively. There was a significant difference in the DCR of PC and PTX (p = 0.0143).

**Table 2:** Best of response of SCT(PTX vs PTX + Cmab).

	all (n = 52)		PTX group (n = 7)		PTX + Cmab group (n = 45)		p-value
	n	%	n	%	n	%	
**CR**	1	1.9	0	0	1	2.2	
**PR**	26	50	3	42.9	23	51.1	
**SD**	18	34.6	1	14.3	17	37.8	
**PD**	7	13.5	3	42.9	4	8.9	
**ORR**	27	51.9	3	42.9	24	53.3	0.606
**DCR**	45	86.5	4	57.1	41	91.1	0.0143

CR, complete response; Cmab, cetuximab; DCR, disease control rate; ORR, objective response rate; PD, progressive disease; PR, partial response; PTX, paclitaxel; SCT, salvage chemotherapy; SD, stable disease.

The Kaplan–Meier survival curves of the 52 patients are presented in Fig 1A and 1B. The estimated median OS and PFS from the first dose of SCT following the initiation of nivolumab therapy were 11.9 months and 7.4 months, respectively. The Kaplan–Meier curves for the OS and PFS were significantly better in the PC than in the PTX group (Fig 1C and 1D).

### Adverse events

All-grade AEs and grade 3/4 AEs were observed in 48 patients (92.3%) and 30 patients (57.7%), respectively, during SCT (S2 Table). Grade 3/4 bone marrow suppression occurred in 15 (28.8%), grade 3 rash in 7 (13.5%), and grade 3/4 hypomagnesemia in 5 (9.6%) patients. No drug-related deaths were reported. There were no significant differences of the occurrence rates of AEs between the PC group and the PTX group.

**Fig 2:**
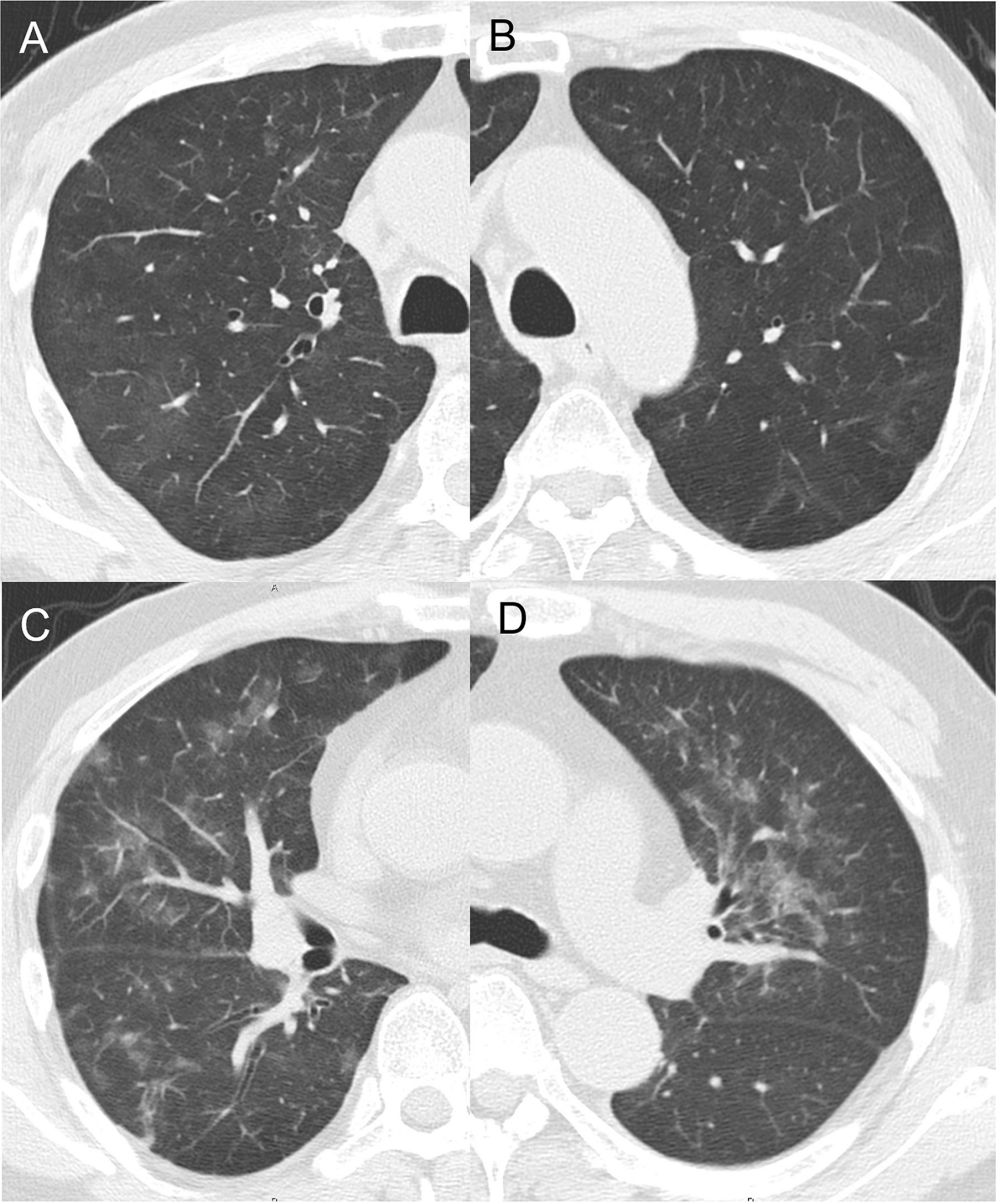
CT imaging of two patients with DI-IP. (A, B) A 67-year-old male patient with hypopharyngeal carcinoma after definitive CRT for primary disease with developing axillary lymph node metastasis. After PD with nivolumab, the patient underwent nine cycles of PC therapy. He was asymptomatic at the time of the CT scan, which was performed to assess the effectiveness of his treatment, and his KL-6 level was elevated to 550 U/ml (reference range: <500U/ml). However, later developed symptoms of dyspnea of the patient and was diagnosed as grade 2 DI-IP. The patient improved with follow-up. (C, D) A 57-year-old male patient with maxillary carcinoma after curative CRT for the primary disease developed localized recurrence where radical excision was impossible. After PD with nivolumab, he underwent nine cycles of PC therapy. He developed respiratory symptoms, such as fever and cough, and a CT scan to investigate the source of inflammation showed non-distracting frosted glass shadows in both lung fields. The serum KL-6 level was elevated to 860 U/ml. The patient was diagnosed as grade 3 DI-IP. He was treated with steroid pulse therapy and improved. CT, computerized tomography; DI-IP, drug-induced interstitial pneumonia; CRT, concurrent chemoradiotherapy; PD, progressive disease.

Regarding the pulmonary disorder, DI-IP diagnosis was made by several experienced physicians and/or a respiratory physician according to the patient’s symptoms; physical examination findings; serum data, such as elevated interstitial pneumonia marker KL-6; and CT images. CT imaging findings of two patients with DI-IP during SCT are presented in Fig 2. Eleven patients (21.2%) were diagnosed as DI-IP (One case in PTX and 10 in PC). Grade 3 DI-IP was observed in three of 11 cases. In these pulmonary disorder cases, SCT was discontinued to avoid DI-IP progression.

### Clinical influence of Cmab administration before ICIs

**Fig 3:**
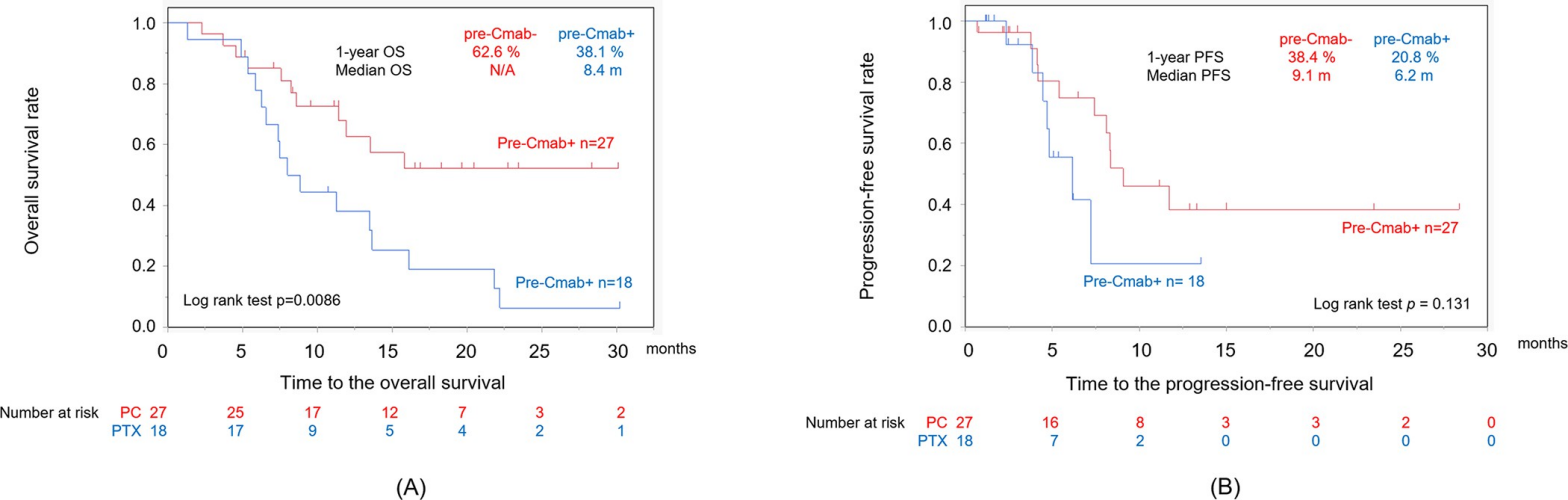
Kaplan–Meier curves for the overall survival (A) and the progression-free survival (B) in R/M HNSCC patients after PTX + Cmab following nivolumab. (A) The overall survival of patients who had received Cmab prior to nivolumab was significantly inferior to that of patients who had not received Cmab before treatment (log-rank test, p = 0.0086). (B) The progression-free survival of patients treated with Cmab before nivolumab was not significantly inferior to that of patients who did not received Cmab before treatment. Cmab, cetuximab; PTX, paclitaxel; R/M HNSCC, recurrent or metastatic head and neck squamous cell carcinoma.

To evaluate the effectiveness and safety of PC following ICIs affected by prior cetuximab use, the PC group was divided into following groups; patients who received Cmab as a palliative therapy before ICIs (pre-Cmab group, Group A) and patients who did not received Cmab before ICIs (no-Cmab group, Group B) (S3 Table). The SCT in group A was above the second line of treatment, which of course was higher than the treatment line of SCT in group B. Furthermore, the number of patients with a PS of 2 or 3 was significantly higher in Group A (p = 0.0307) than those in Group B. There was no differences in ORR, DCR or PFS among Group A and B (Table 3, Fig 3). However, Group B had a better OS than Group A. on the other hand,the incidence of DI-IP was 10 out of 45 cases (22.2%), and eight cases were grade 1/2. Although there were no statistically significant differences, eight Of 10 DI-IP cases were in Group B (Table 4).

**Table 3:** Best response of SCT (paclitaxel and cetuximab).

	Group A: Cmab before ICI (n = 18)		Group B: Never Cmab before ICI (n = 27)		p-value
	n	%	n	%	
**CR**	0	0.0	1	3.7	
**PR**	8	44.4	15	55.6	
**SD**	8	44.4	9	33.3	
**PD**	2	11.1	2	7.4	
**ORR**	8	44.4	16	59.3	0.329
**DCR**	16	88.9	25	92.6	0.669

CR, complete response; Cmab, cetuximab; DCR, disease control rate; ICI, immune checkpoint inhibitor; ORR, objective response rate; PD, progressive disease; PR, partial response; SCT, salvage chemotherapy; SD, stable disease.

**Table 4:** Profiles of AEs that appeared during SCT (paclitaxel and cetuximab; Group A vs. Group B).

Category	Group A: Cmab before ICI (n = 18)				Group B: Never Cmab before ICI (n = 27)				
	Total (n)	(%)	Grade ≥3 (n)	(%)	Total (n)	(%)	Grade ≥3 (n)	(%)	p-value (all / Grade ≥3)
**Any**	15	83.3	10	55.6	27	100.0	16	59.3	0.0281/0.805
**SKIN**	6	33.3	4	22.2	20	74.1	5	18.5	0.0067/0.761
Rash	4	22.2	3	16.7	19	70.4	4	14.8	0.0015/0.867
Perionychia	4	22.2	2	11.1	11	40.7	1	3.7	0.197/0.329
**Gastrointestinal**	4	22.2	0	0.0	10	37.0	1	3.2	0.140/0.409
**Pulmonary**	3	16.7	1	5.6	9	33.3	2	6.5	0.216/0.803
Interstitial pneumonitis	2	11.1	1	5.6	8	29.6	1	3.2	0.143/0.768
Bacterial pneumonia	1	5.6	0	0.0	1	3.7	1	3.2	0.768/0.409
**Fatigue**	5	27.8	0	0.0	6	22.2	1	3.2	0.467/0.600
**Periferal neuropathy**	1	5.6	0	0.0	7	25.9	0	0.0	0.0800/-
**Fever increase**	1	5.6	0	0.0	4	14.8	1	3.2	0.333/0.409
**Leukopenia**	9	50.0	6	33.3	17	60.7	9	33.3	0.388/0.624
**Neutropenia**	9	50.0	5	27.8	15	55.6	6	19.4	0.714/0.671
**Anemia**	8	44.4	3	16.7	14	51.9	0	0.0	0.626/0.0281
**Hypomagnesia**	8	44.4	3	16.7	9	33.3	2	6.5	0.451/0.333
**Others**	4	22.2	1	5.6	8	29.6	5	18.5	
Thromboembolism	0	0.0	0	0.0	0	0.0	0	0.0	
Endotracheal hemorrhage	0	0.0	0	0.0	1	3.7	1	3.2	
Catheter-related infection	1	5.6	1	5.6	0	0.0	0	0.0	
Edema limbs	1	5.6	1	5.6	1	3.7	0	0.0	
Hyperglycemia	0	0.0	0	0.0	1	3.7	1	3.2	
Hyperamylasemia	0	0.0	0	0.0	1	3.7	1	3.2	
Liver dysfunction	1	5.6	0	0.0	1	3.7	0	0.0	
Renal dysfunction	1	5.6	0	0.0	1	3.7	0	0.0	
Electrolyte disorder	0	0.0	0	0.0	2	7.4	0	0.0	
Hypoalbuminemia	1	5.6	0	0.0	0	0.0	0	0.0	
Dysgeusia	0	0.0	0	0.0	1	3.7	0	0.0	

AE, adverse event; Cmab, cetuximab; ICI, immune checkpoint inhibitor; SCT, salvage chemotherapy

## Discussion

The increasing focus on sequential therapies in the palliative treatment of R/M HNSCC and the therapeutic efficacy of SCT after ICI therapy has been attracting attention. On the other hand, the development of DI-IP, which is a common AE in Japanese patients, cannot be ignored [14]. Especially, previous studies reported that the administration of EGFR tyrosine kinase inhibitors (TKIs) increased the risk of interstitial lung disease after treatment with ICI [15]. Therefore, evaluating the efficacy and safety of the combined use of anti-EGFR antibody, cetuximab, and PTX after immunotherapy in R/M SCCHN is important.

The effectiveness of nivolumab in this group of patients was similar to that in previous reports on R/M HNSCC [16–18]. Furthermore, the therapeutic response of SCT after our nivolumab treatment was also favorable. In regard to the treatment effect of PC, Hitt et al. reported in their phase Ⅱ study that 46 patients with R/M HNSCC who were treated with PC as the first-line therapy had an ORR and DCR of 54% and 80% and PFS and OS of 4.2 months and 8.1 months, respectively [19]. In our current study, the ORR and DCR in the patients treated with PC as SCT were 53.3% and 91.1%, and the estimated median OS and PFS from the first dose of PC were 13.5 months and 8.1 months, respectively. These results were favorable compared to those of the Hitt trial and consistent with the previous analysis. Combination therapies of cetuximab with PTX as SCT were proven to be effective even in a later-line setting [19, 20]. In our results, comparing PTX and PC therapy as a treatment for SCT, PC therapy appeared to be more effective. However, only seven patients participated in the PTX group, and the effectiveness of this treatment should be carefully examined. Guiard et al. reported an ORR of 39.4% in a much larger group of 71 patients treated with nivolumab followed by PTX, which is similar to our results in the PTX group [21]. Of course, we could not make a simple comparison, however our PC group shows a higher therapeutic effect, and we consider the possibility that the PC group is more effective than the PTX group in SCT. Basic research also supports the superiority of therapeutic efficacy of immunotherapy followed by chemotherapy over chemotherapy followed by immunotherapy [22, 23]. Anticancer drugs work by reducing the number of Treg cells and reactivating cytotoxic T cells, thereby suppressing immune tolerance in cancer [22]. Additionally, serum programmed cell death protein 1 (PD-1) antibodies are halved in 12–20 days, while PD-1 binding may produce a concomitant effect when anticancer drugs are administered after nearly 2 months of continuous immunotherapy [23]. Consistent with previous reports, the overall treatment effect of SCT in this study was favorable [16, 17, 24].

We found a difference in the prolongation of OS from SCT initiation in patients without Cmab administration before ICIs, although there was no significant difference in ORR, DCR, and PFS. In addition, Both OS and PFS of SCT were inferior in the patients who received Cmab as a palliative therapy before ICIs compared to those who did not received Cmab before ICIs. Therefore, the results were expected to be highly effective for treatment as SCT after ICIs without prior Cmab administration. We previously elucidated that low pre-SCT CRP levels and a low pre-SCT neutrophil to lymphocyte ratio predicted a better prognosis after SCT in patients with R/M HNSCC [8]. Ueki Y et al. also reported that SCT is more effective in treatment if there is more expression of positive programmed death-ligand 1 in the tumor [25]. Likewise, the presence or absence of Cmab pretreatment may also be used as a predictor of treatment response in SCT.

Although we have reported that the therapeutic effect of SCT is excellent, the incidence of DI-IP is high [6, 11]. Compared with our previous reports, our current cohort study included more cases of prior Cmab administration. As a result, we found that DI-IP occurred in 10 (22.2%) of all 45 cases who received PC. Three (16.7%) of 18 patients were in the pre-Cmab group developed DI-IP, whereas 9 (33.3%) of 27 patients were in the group that received Cmab after the ICI treatment. Thus, our findings suggested that PC therapy after ICI treatment without pre-Cmab treatment might have a higher risk of inducing DI-IP. However, many cases were classified as a grade 1 AE (solely abnormal laboratory results without symptoms). It is important to detect early DI-IP and to prevent DI-IP from worsening by promptly interrupting treatment and administering steroids. In our institution, we usually provide short-term educational hospitalization for patients who receive their first dose of ICI or SCT. They also check the serum level of KL-6 once a month. If there is any suspicion of DI-IP, the patient undergoes CT imaging and receives a prompt consultation with a respiratory physician. Regarding other side effects, the incidence of skin disorders was also lower in the Cmab pre-treatment group, suggesting the possibility of selection bias due to the presence or absence of pre-Cmab treatment. On the other hand, overall adverse events in patients treated with PTX and Cmab tended to be higher than previously reported [19, 26]. Enokida et al. reported Grade 3 adverse events of leukopenia (24%), neutropenia (13%), anemia (13%), and hypomagnesemia (13%) with weekly PTX + Cmab as palliative treatment after CRT, which were generally less frequent than in this study [26]. The difference in toxicity due to treatment being limited to the 1st line therapy and PTX being administered for only 3 of the 4 weeks in their regimen may have affected adverse events. However, no grade 5 events occurred in our study, suggesting that adverse events can be managed appropriately. In general, PC therapy as a highly effective SCT could be safely implemented.

This retrospective study had some limitations. First, a limited number of patients were included, of whom 7 of the 52 patients received PTX only. Patients who have received Cmab and had serious AEs are unlikely to receive Cmab-containing chemotherapy as SCT. Thus, these factors may have contributed to the bias. In the comparison of the PC group with and without prior Cmab administration, the chemotherapy line was higher in the group with prior Cmab administration, which may also affect prognosis.

This is the first study to compare the clinical characteristics of patients who received PC and PTX as SCT after ICIs as palliative treatment of R/M HNSCC. We found that the therapeutic efficacy of the PC regimen as SCT was high and was even higher in those patients who had not received Cmab prior to ICI treatment. On the other hand, the incidence of DI-IP (mainly grades 1 and 2) tended to be higher in the group without prior Cmab administration. Both clinical findings and the serum level of KL-6 value are essential to detect DI-IP. Furthermore, DI-IP may be asymptomatic, so CT imaging should be immediately performed if DI-IP is suspected. Our study findings will help to guide decision making on the choice of chemotherapy and whether to continue treatment, switch to other agents, or move to best supportive care-only treatment. Furthermore, our findings may help to improve the treatment strategy for palliative chemotherapy of R/M HNSCC patients. The potential prognostic impact of these markers needs further investigation using prospective studies.

## Conclusion

Excellent therapeutic efficacy can be expected with PC therapy after ICI administration for R/M HNSCC patients. However, caution must be exercised because the incidence of DI-IP may be higher in patients without prior Cmab treatment.

## Supporting information

S1 Table Best of response of ICI. (DOCX)

S2 Table Profiles of AEs appeared during SCT in all patients. (DOCX)

S3 Table Characteristics of the patients in Groups A and B.
(DOCX)

S1 Dataset (XLSX)
