# Correction: Effectiveness and safety of weekly paclitaxel and cetuximab as a salvage chemotherapy following immune checkpoint inhibitors for recurrent or metastatic head and neck squamous cell carcinoma: a multicenter clinical study

**DOI:** 10.1371/journal.pone.0303720

**Published:** 2024-05-09

**Authors:** Takahiro WakasakiI, Tomomi Manako, Ryuji YasumatsuI, Hirotaka Hara, Satoshi Toh, Muneyuki Masuda, Moriyasu YamauchiID, Yuichiro Kuratomi, Emi Nishimura, Toranoshin Takeuchi, Mioko Matsuo, Rina Jiromaru, Kazuki Hashimoto, Noritaka Komune, Takashi Nakagawa

There are errors in the author affiliations. The correct affiliations are as follows:

Takahiro Wakasaki **1**, **2**, Tomomi Manako **1**, Ryuji Yasumatsu **1**,**3** *, Hirotaka Hara **4**, Satoshi Toh **4**, Muneyuki Masuda **4**, Moriyasu Yamauchi **5**, Yuichiro Kuratomi **5**, Emi Nishimura **6**, Toranoshin Takeuchi **6**, Mioko Matsuo **1**, Rina Jiromaru **1**, Kazuki Hashimoto **1**, Noritaka Komune **1**, and Takashi Nakagawa **1**

**1** Department of Otorhinolaryngology, Graduate School of Medical Sciences, Kyushu University, Fukuoka 812–8582, Japan **2** Department of Otorhinolaryngology, Japanese Red Cross Fukuoka Hospital, Fukuoka, 815–8555, Japan. **3** Department of Otorhinolaryngology-Head and Neck Surgery, Faculty of Medicine, Kindai University, Osaka 589–8511, Japan. **4** Department of Head and Neck Surgery, National Hospital Organization, Kyushu Cancer Center, Fukuoka 812–8582, Japan **5** Department of Otolaryngology, Head and Neck Surgery, Saga University Hospital, Saga, 849–8501, Japan **6** Department of Otorhinolaryngology, Kitakyushu Municipal Medical Center, Kitakyushu 802–8561, Japan
