# Supplementary material for: Effectiveness and safety of weekly paclitaxel and cetuximab as a salvage chemotherapy following immune checkpoint inhibitors for recurrent or metastatic head and neck squamous cell carcinoma: A multicenter clinical study
Source: PLoS One. 2022 Jul 28;17(7):e0271907. doi: 10.1371/journal.pone.0271907 (PMC9333293; doi:10.1371/journal.pone.0271907)
Supplement: S1 Table — (DOCX) [file pone.0271907.s001.docx]

S1 Table. Best of Response of ICI

|  | **All (n=52)** | |
| --- | --- | --- |
|  | **n** | **%** |
| **CR** | 0 | 0 |
| **PR** | 9 | 17.3 |
| **SD** | 19 | 36.5 |
| **PD** | 23 | 44.2 |
| **N/A** | 1 | 1.9 |
| **ORR** | 9 | 17.3 |
| **DCR** | 28 | 53.8 |

CR, complete response; DCR, disease control rate; ICI, immune checkpoint inhibitor; N/A, not available; ORR, objective response rate; PD, progressive disease; PR, partial response; SD, stable disease.
