# Supplementary material for: Effectiveness and safety of weekly paclitaxel and cetuximab as a salvage chemotherapy following immune checkpoint inhibitors for recurrent or metastatic head and neck squamous cell carcinoma: A multicenter clinical study
Source: PLoS One. 2022 Jul 28;17(7):e0271907. doi: 10.1371/journal.pone.0271907 (PMC9333293; doi:10.1371/journal.pone.0271907)
Supplement: S2 Table — (DOCX) [file pone.0271907.s002.docx]

S2 Table. Profiles of AEs appeared during SCT in all patients

| **Category** | **All patients (n=52)** | | | | **PTX (n=7)** | | | | **PTX + Cmab (n=45)** | | | | **p-value  (All / Grade ≥3)** |
| --- | --- | --- | --- | --- | --- | --- | --- | --- | --- | --- | --- | --- | --- |
|  | **Total (n)** | **(%)** | **Grade ≥3 (n)** | **(%)** | **Total (n)** | **(%)** | **Grade ≥3 (n)** | **(%)** | **Total (n)** | **(%)** | **Grade ≥3 (n)** | **(%)** |  |
| **Any** | 48 | 92.3 | 30 | 57.7 | 6 | 85.7 | 4 | 57.1 | 42 | 93.3 | 26 | 57.8 | 0.480/0.975 |
|  |  |  |  |  |  |  |  |  |  |  |  |  |  |
| **SKIN** | 29 | 55.8 | 12 | 23.1 | 3 | 42.9 | 3 | 42.9 | 26 | 57.8 | 9 | 20.0 | 0.460/0.182 |
| Rash | 26 | 50.0 | 8 | 15.4 | 3 | 42.9 | 1 | 14.3 | 23 | 51.1 | 7 | 15.6 | 0.685/0.931 |
| Perionychia | 17 | 32.7 | 5 | 9.6 | 2 | 28.6 | 2 | 28.6 | 15 | 33.3 | 3 | 6.7 | 0.803/0.0674 |
|  |  |  |  |  |  |  |  |  |  |  |  |  |  |
| **Gastrointestinal** | 16 | 30.8 | 1 | 1.9 | 3 | 42.9 | 0 | 0.0 | 13 | 28.9 | 1 | 2.2 | 0.456/0.690 |
|  |  |  |  |  |  |  |  |  |  |  |  |  |  |
| **Pulmonary** | 13 | 25.0 | 4 | 7.7 | 1 | 14.3 | 1 | 14.3 | 12 | 26.7 | 3 | 6.7 | 0.482/0.482 |
| Interstitial pneumonitis | 11 | 21.2 | 3 | 5.8 | 1 | 14.3 | 1 | 14.3 | 10 | 22.2 | 2 | 4.4 | 0.632/0.299 |
| Bacterial pneumonia | 2 | 3.8 | 1 | 1.9 | 0 | 0.0 | 0 | 0.0 | 2 | 4.4 | 1 | 2.2 | 0.570/0.690 |
|  |  |  |  |  |  |  |  |  |  |  |  |  |  |
| **Fatigue** | 11 | 21.2 | 1 | 1.9 | 0 | 0.0 | 0 | 0.0 | 11 | 24.4 | 1 | 2.2 | 0.141/0.690 |
|  |  |  |  |  |  |  |  |  |  |  |  |  |  |
| **Periferal neuropathy** | 10 | 19.2 | 0 | 0.0 | 2 | 28.6 | 0 | 0.0 | 8 | 17.8 | 0 | 0.0 | 0.500/- |
|  |  |  |  |  |  |  |  |  |  |  |  |  |  |
| **Fever increase** | 5 | 9.6 | 1 | 1.9 | 0 | 0.0 | 0 | 0.0 | 5 | 11.1 | 1 | 2.2 | 0.354/0.690 |
|  |  |  |  |  |  |  |  |  |  |  |  |  |  |
| **Leukopenia** | 38 | 73.1 | 15 | 28.8 | 1 | 14.3 | 1 | 14.3 | 37 | 82.2 | 14 | 31.1 | 0.0322/0.310 |
|  |  |  |  |  |  |  |  |  |  |  |  |  |  |
| **Neutropenia** | 25 | 48.1 | 11 | 21.2 | 1 | 14.3 | 0 | 0.0 | 24 | 53.3 | 11 | 24.4 | 0.0544/0.141 |
|  |  |  |  |  |  |  |  |  |  |  |  |  |  |
| **Anemia** | 25 | 48.1 | 4 | 7.7 | 2 | 28.6 | 1 | 14.3 | 23 | 51.1 | 3 | 6.7 | 0.316/0.482 |
|  |  |  |  |  |  |  |  |  |  |  |  |  |  |
| **Hypomagnesia** | 18 | 34.6 | 5 | 9.6 | 1 | 14.3 | 0 | 0.0 | 17 | 37.8 | 5 | 11.1 | 0.224/0.354 |
|  |  |  |  |  |  |  |  |  |  |  |  |  |  |
| **Others** | 17 | 32.7 | 7 | 13.5 | 4 | 57.1 | 2 | 28.6 | 12 | 26.7 | 7 | 15.6 |  |
| Thromboembolism | 1 | 1.9 | 1 | 1.9 | 1 | 14.3 | 1 | 14.3 | 0 | 0.0 | 0 | 0.0 |  |
| Endotracheal hemorrhage | 1 | 1.9 | 1 | 1.9 | 0 | 0.0 | 0 | 0.0 | 1 | 2.2 | 1 | 2.2 |  |
| Catheter-related infection | 2 | 3.8 | 2 | 3.8 | 0 | 0.0 | 1 | 14.3 | 2 | 4.4 | 1 | 2.2 |  |
| Edema limbs | 2 | 3.8 | 1 | 1.9 | 2 | 28.6 | 0 | 0.0 | 0 | 0.0 | 1 | 2.2 |  |
| Hyperglycemia | 1 | 1.9 | 1 | 1.9 | 1 | 14.3 | 0 | 0.0 | 0 | 0.0 | 1 | 2.2 |  |
| Hyperamylasemia | 1 | 1.9 | 1 | 1.9 | 0 | 0.0 | 0 | 0.0 | 1 | 2.2 | 1 | 2.2 |  |
| Liver dysfunction | 3 | 5.8 | 0 | 0.0 | 0 | 0.0 | 0 | 0.0 | 3 | 6.7 | 0 | 0.0 |  |
| Renal dysfunction | 3 | 5.8 | 0 | 0.0 | 0 | 0.0 | 0 | 0.0 | 3 | 6.7 | 0 | 0.0 |  |
| Electrolyte disorder | 3 | 5.8 | 0 | 0.0 | 0 | 0.0 | 0 | 0.0 | 3 | 6.7 | 0 | 0.0 |  |
| Hypoalbuminemia | 1 | 1.9 | 0 | 0.0 | 0 | 0.0 | 0 | 0.0 | 1 | 2.2 | 0 | 0.0 |  |
| Dysgeusia | 1 | 1.9 | 0 | 0.0 | 0 | 0.0 | 0 | 0.0 | 1 | 2.2 | 0 | 0.0 |  |

AE, adverse event; Cmab, cetuximab; ICI, immune checkpoint inhibitor; PTX, paclitaxel; SCT, salvage chemotherapy
