# Supplementary material for: Effectiveness and safety of weekly paclitaxel and cetuximab as a salvage chemotherapy following immune checkpoint inhibitors for recurrent or metastatic head and neck squamous cell carcinoma: A multicenter clinical study
Source: PLoS One. 2022 Jul 28;17(7):e0271907. doi: 10.1371/journal.pone.0271907 (PMC9333293; doi:10.1371/journal.pone.0271907)
Supplement: S3 Table — Characteristics of the patients in Groups A and B. (DOCX) [file pone.0271907.s003.docx]

S3 Table Characteristics of the patients in Groups A and B

|  | **Group A: Cmab before ICI (n = 18)** | | **Group B: Never Cmab before ICI (n =27)** | |
| --- | --- | --- | --- | --- |
|  | **n** | **%** | **n** | **%** |
| **Median Age** |  | 65 (33–79) |  | 67 (45–79) |
| ≥65 years old | 11 | 61.1 | 14 | 51.9 |
| <65 years old | 7 | 38.9 | 13 | 48.1 |
| **Gender** |  |  |  |  |
| Male | 15 | 83.3 | 23 | 85.2 |
| Female | 3 | 16.7 | 4 | 14.8 |
| **Smoking status** |  |  |  |  |
| Smoker | 13 | 72.2 | 24 | 88.9 |
| Never | 5 | 27.8 | 3 | 11.1 |
| **ECOG PS at the first administration of SCT** |  |  |  |  |
| PS 0–1 | 13 | 72.2 | 26 | 96.3 |
| PS 2-3 | 5 | 27.8 | 1 | 3.7 |
| **Primary site** |  |  |  |  |
| Oral | 2 | 11.1 | 5 | 18.5 |
| Nasopharynx | 7 | 38.9 | 6 | 22.2 |
| Oropharynx | 0 | 0.0 | 2 | 7.4 |
| Hypopharynx | 1 | 5.6 | 4 | 14.8 |
| Larynx | 4 | 22.2 | 7 | 25.9 |
| Sinonasal tract | 3 | 16.7 | 1 | 3.7 |
| Others | 1 | 5.6 | 2 | 7.4 |
| **Disease State** |  |  |  |  |
| LA | 9 | 50.0 | 8 | 29.6 |
| DM | 5 | 27.8 | 10 | 37.0 |
| LA + DM | 4 | 22.2 | 9 | 33.3 |
| **Administration line of SCT in palliative therapy** |  |  |  |  |
| 2nd | 0 | 0.0 | 26 | 96.3 |
| 3rd | 14 | 77.8 | 1 | 3.7 |
| 4th | 3 | 16.7 | 0 | 0.0 |
| 5th | 1 | 5.6 | 0 | 0.0 |
| **Cause of cessation of Nivolumab** |  |  |  |  |
| PD | 26 | 96.3 | 16 | 88.9 |
| AE | 1 | 3.7 | 0 | 0.0 |
| PD+AE | 0 | 0.0 | 0 | 0.0 |
| Other reason | 0 | 0.0 | 2 | 11.1 |
| **Cause of cessation of SCT** |  |  |  |  |
| PD | 9 | 33.3 | 7 | 38.9 |
| AE | 12 | 44.4 | 10 | 55.6 |
| PD+AE | 1 | 3.7 | 0 | 0.0 |
| Other reason | 1 | 3.7 | 0 | 0.0 |
| Ongoing | 2 | 7.4 | 1 | 5.6 |

AE, adverse event; Cmab, cetuximab; DM, distant metastasis; PS, performance status; ECOG, Eastern Cooperative Oncology Group; ICI, immune checkpoint inhibitor; LA, locally advanced disease; PD, progressive disease; PS, performance status; SCT, salvage chemotherapy
